# A Full Bayesian Approach for Boolean Genetic Network Inference

**DOI:** 10.1371/journal.pone.0115806

**Published:** 2014-12-31

**Authors:** Shengtong Han, Raymond K. W. Wong, Thomas C. M. Lee, Linghao Shen, Shuo-Yen R. Li, Xiaodan Fan

**Affiliations:** 1 Department of Statistics, The Chinese University of Hong Kong, Shatin, N.T., Hong Kong, China; 2 Department of Statistics, Iowa State University, Ames, IA, United States of America; 3 Department of Statistics, University of California Davis, Davis, CA, United States of America; 4 Department of Information Engineering, The Chinese University of Hong Kong, Shatin, N.T., Hong Kong, China; 5 University of Electronic Science and Technology of China, Chengdu, China; University of Miami, United States of America

## Abstract

Boolean networks are a simple but efficient model for describing gene regulatory systems. A number of algorithms have been proposed to infer Boolean networks. However, these methods do not take full consideration of the effects of noise and model uncertainty. In this paper, we propose a full Bayesian approach to infer Boolean genetic networks. Markov chain Monte Carlo algorithms are used to obtain the posterior samples of both the network structure and the related parameters. In addition to regular link addition and removal moves, which can guarantee the irreducibility of the Markov chain for traversing the whole network space, carefully constructed mixture proposals are used to improve the Markov chain Monte Carlo convergence. Both simulations and a real application on cell-cycle data show that our method is more powerful than existing methods for the inference of both the topology and logic relations of the Boolean network from observed data.

## Introduction

A central focus in genomic research is to infer how genes are related to each other. Due to the complexity of real biological systems, it is essential to learn genetic networks in a holistic rather than an atomistic manner [1]. Various network models have been proposed to describe gene regulatory mechanisms, such as deterministic Boolean networks, random Boolean networks [2], probabilistic Boolean networks [3], probabilistic gene regulatory networks [4], Bayesian networks [5], [6], etc. For a review of methods for reconstructing genetic networks, see [7]. Each model has its own advantages and drawbacks. Boolean networks have the appealing characteristics of model simplicity, dynamic complexity and robustness to the noisy data. Moreover, recent research indicates that many realistic biological questions can be answered by the simple Boolean formulation, which essentially emphasizes fundamental and generic principles rather than quantitative biochemical details [8]. Biologists also traditionally prefer using ON and OFF to describe gene expression status. However, Boolean networks suffer the risk of losing useful information because of the two-state simplification for the continuous gene expression values. A detailed discussion of the prospects and limitations of Boolean genetic network models can be found in [9].

A number of algorithms have been proposed to infer Boolean genetic networks from observed data sets; [10] provided a good review of these algorithms. In [11], two popular algorithms, REVEAL [12] and Best-Fit Extension (BFE) [13], are implemented in a R package called BoolNet. REVEAL is based on exhaustive mutual information comparison, but it essentially assumes a deterministic Boolean network model. Thus it is not always able to reconstruct networks in the presence of noisy and inconsistent measurements in the input data. BFE accommodates noisy input data by minimizing the number of misclassifications. Its optimization is performed for each output node separately instead of for the whole network jointly. More recently, [14] proposed a likelihood-based approach to reconstruct Time Delay Boolean Networks (TDBN) from noisy data, but again the likelihood is maximized for each output node separately. To achieve better inference efficiency and accuracy, there is a need of new network reconstruction methods which use the optimization of a proper objective function simultaneously for the whole network. In this paper, we developed a full Bayesian Inference approach for a Boolean Network (BIBN), which is based on maximizing the joint posterior probability over the whole network. We show the new BIBN method outperforms REVEAL [12], BFE [13] and TDBN [14] through simulation. We also applied BIBN on the yeast cell-cycle data.

## Materials and Methods

### Model

Our method uses a probabilistic Boolean network model, where each node represents a gene with binary expression values. More specifically, we model the relations among the 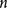 genes under study as a directed acyclic graph denoted by a set of components 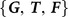, where 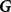 represents the set of nodes 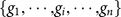, 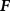 denotes a set of Boolean functions 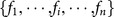, and 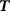 represents the topology of the network, i.e., the input-output connectivity information. Here 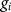 denotes both the node corresponding to the 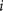-th gene and its gene expression values. Suppose we have 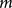 observations of the network, then 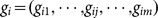. Each value 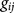 is a binary variable, taking values from 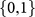. The binary formulation corresponds to the simplification of the gene activity to either an active (ON) or inactive (OFF) state. The set of input nodes of the node 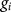, denoted as its parent set 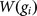, is the set of genes which may directly affect the gene expression 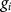. The information about 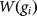 is derived from the topology 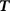. The Boolean function 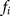 is composed of four commonly used logic operators: 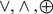 (representing AND, OR, exclusive-OR respectively) and the logic 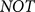 operation (the 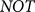 operation on 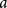 is denoted by 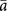).

If 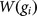 is an empty set, it means the 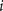-th gene is not regulated by any other genes in the network. In this case, we call 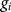 as a root node, and assume an independent Bernoulli distribution for it, i.e., 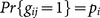 and 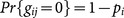.

If 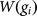 is non-empty, we assume that 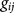 is determined by 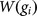 through 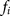 and an independent and identically distributed (i.i.d.) additive noise 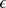, which follows a Bernoulli distribution, i.e.:
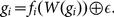
(1)


If the 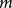 observations of the network are independent from each other, 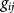 is determined by the 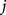-th observation of its parent set 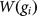. If the 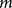 observations of the 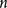 genes form a synchronized time series, 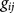 shall be determined by the 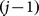-th observation of its parent set 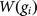. In either case, the noise term 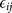 of 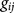 is assumed to be independent and identically distributed (i.i.d.) with 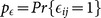 and 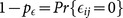. For presentation convenience, we will stick to the notations as if the 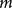 observations are independent, although our algorithm suits both cases.

Assume the network contains 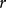 root nodes and, for notation convenience, assume the root nodes are 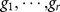. Denote 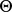 as the set of the noise parameter 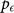 and all of the 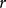 root node parameters 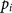. We can then write down the full likelihood of the model as: 
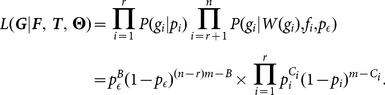
(2)


Here 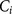 represents the number of non-zero data points 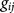 of the root node 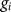, and 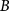 represents the total number of non-root data points 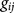 which is not equal to 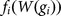. That is, 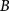 counts the number of times that 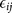 is equal to 1. The full likelihood is consisted of two parts. The first part is contributed by the noise and the second part is from all root nodes.

The number of input nodes of 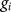 is referred to as the in-degree of 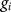. The computing complexity will inevitably increase if the in-degree increases, although the principle of our algorithm suits networks with any in-degree. Similar to BFE [13] and TDBN [14], we will focus on the case where the maximum in-degree of all nodes in the network is bounded by 2. Therefore, both the number of valid network topologies (defined now as all directed acyclic graphs of 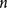 nodes where every node has no more than 2 input nodes) and the number of possible Boolean function types for 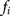 are also bounded. Although this in-degree constraint is rooted in the computing scalability, it actually has biological justifications because most genes in the cell are regulated by only a very small number of genes [15]–[17]. It is believed that most Boolean functions require few essential variables [18] and networks where most nodes have many parents will offer little scientific insight [19].

In this paper, we are interested in inferring the network topology 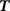 and Boolean functions 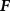 based on 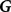, i.e., 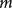 observations of the 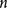 concerned genes.

### Algorithm

To fit the above models to input data sets, we use a full Bayesian approach to take advantage of the conditionally independent nature of some random variables in the network model, to take account of the estimation uncertainty and to provide a convenient way to incorporate prior knowledge. Markov chain Monte Carlo (MCMC) algorithms will be developed to sample from the joint posterior distribution of the network topology and Boolean functions, which will provide both a point estimate and an uncertainty measure for these unknown variables.

#### Prior Distributions

For Bayesian inference, we need to specify the prior distributions for 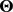, 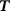 and 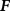. If we have some prior knowledge about these unknown variables, it is an advantage of the Bayesian approach to seamlessly integrate this knowledge into the inference result. If we do not have any prior knowledge, specifying a flat prior will result in a posterior inference which is equivalent to the maximum likelihood estimation. Overall, we assume 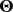 is independent of 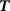 and 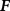 in the prior distribution, i.e., 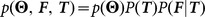.

For all 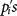 and 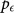 in 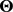, we assume that they follow independent Beta distributions as in [20]–[23]. More specifically, we assume that the noise parameter 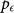 is sampled from 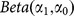, and all root parameters 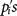 are independently sampled from 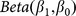. The parametric form of Beta distribution will make the computation more convenient since it is the conjugate prior for the likelihood. The hyper-parameters 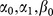 and 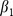 are chosen constants. Since we know little about 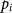, we can set 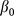 and 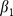 as 1, which will result in a flat prior distribution. As the noise rate 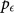 should not be too big, we set 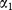 to be smaller than 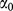.

Let 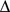 denote the total number of valid network topology as defined before. We use uniform prior for 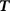, i.e., 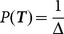.

As for 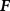, the actual number of possible function forms for 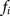 is dependent on the topology 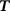 and is no more than 16 if the maximum in-degree is 2. For its prior, we assume that 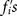 are independent of each other conditional on 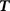 and 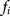 is sampled uniformly from all possible non-degenerative Boolean functions of 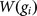. For example, if 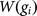 is the set 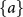, 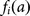 can be either 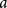 or 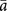; if 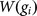 is the set 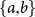, 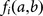 has 10 non-degenerative choices: 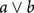, 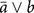, 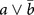, 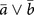, 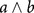, 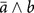, 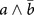, 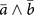, 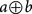, 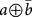.

#### Posterior Distributions

From the above prior distributions and the full likelihood, it is straightforward to derive the following joint posterior distribution: 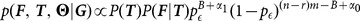


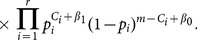
(3)


Since the number of root nodes is unknown and is determined by the topology 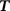, the dimension of 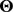 may change once we change the topology. Thus, if we use an MCMC algorithm to directly sample from the above joint posterior distribution, we have to deal with the trans-dimensional problem. Although theoretically some algorithms, such as reversible jump MCMC [24], can be used to handle this problem, the convergence speed of such MCMC algorithms is still problematic. To circumvent this problem, we analytically integrate out all 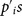 and 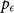 from the above posterior distribution, which results in the following collapsed version of the posterior distribution: 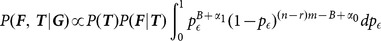


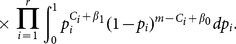
(4)


We have designed an MCMC algorithm to sample from 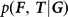, which avoids the dimension change caused by 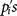. More specifically, we update 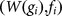 iteratively for all 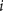 with Metropolis-Hastings (MH) algorithms. If we are also interested in estimating 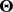, we can subsequently estimate 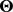 from 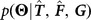 after we obtain the posterior estimates 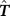 and 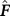.

#### Constructing Efficient Proposal Distributions for MH algorithms

One major concern of using MCMC algorithms to sample from complicated distributions, such as the posterior network topology space, is the convergence rate, which will determine the computing time to achieve a stationary sample of a desired effective sample size. For the MH algorithm which we will use to sample from 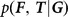, a good proposal distribution is the key for its sampling efficiency. We will first use the 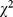 goodness-of-fit test to pick out well-fitted parent sets and corresponding functions for each node as preferential candidates, then construct a node-specific proposal distribution as a mixture of random-walk and weighted sampling from the preferential candidates. These proposal distributions will not change the stationary distribution of the MCMC chain, but it will improve the mixing of the Markov chain by placing more effect on more likely regions of the parameter space.

The 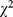 goodness-of-fit test to check how well a combination 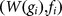 fits the data of 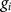 goes as follow. Without loss of generality, considering the two-parent case with 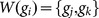 and the OR function 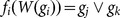. There are 4 possible values for 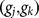, i.e., 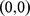, 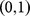, 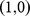, 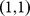. Denote the probabilities of the 4 values as 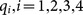, which satisfy 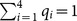. According to the model in Equation 1, the probabilities of the 8 possible values of 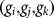 are listed in Table 1, where all unknown parameters will be estimated from the data of 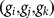. The 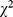 goodness-of-fit test is then used to test whether the observed frequencies of the 8 possible values fit the distribution in Table 1. If fitting, the combination 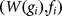 is called a preferential candidate for 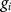. The reciprocal of the noise level estimate 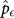 will be used to weigh the preferential candidate.

**Table 1:** The theoretical distribution of 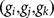 for the relation 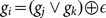.

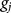	0	0	0	0	1	1	1	1
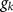	0	0	1	1	0	0	1	1
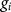	0	1	0	1	0	1	0	1
Probability	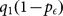	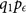	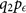	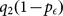	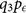	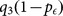	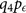	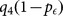

There are 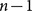 and 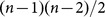 possible choices for 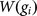, 2 and 10 possible choices for 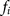, in the case of one parent and two parents, respectively. All possible parent and function combinations are tested in the similar way one by one. The resulted preferential candidates and their associated weights are used to construct two multinomial distributions, one for the one-parent case and one for the two-parent case, which are called the preferential distributions of the node 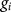. Two uniform distributions are constructed for the node 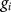 by assigning equal weights to all of its possible parent and function combinations in the case of one parent and two parents, separately. The proposal distribution for updating 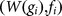 in the case of a given number of parents is the mixture distribution of the corresponding preferential distribution and the corresponding uniform distribution of the node 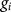, with the mixing proportion of preferential distribution gradually reducing from one to a selected percentage. This proposal constructing procedure is applied to each node.

#### The MCMC Algorithm

The general MCMC framework will be the Metropolis-within-Gibbs algorithm, which starts with initial values of 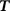 and 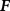, and iteratively updates them from their conditional posterior distributions until the chain is converged.

Updating network topology refers to link addition and removal between nodes, which is equivalent to changing nodes' parent sets. There are three types of MCMC moves to update the parent sets: adding parent(s), removing parent(s) and swapping parent(s). We call one move as legal if it results in a valid network topology as defined previously. For instance, for a node currently without any input node, there may be 2 legal moves, i.e., adding one parent and adding two parents. But if adding parent(s) leads to a cyclic graph, that specific move is illegal.

Once the topology 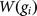 changes, the associated Boolean function 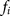 will also have to change. We sequentially and iteratively update each node's parent set 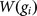 and associated function 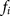 through a MH algorithm using the proposal distributions constructed in the previous subsection.

## Results

### Simulation Studies

Simulation studies are performed to validate our method and compare with existing methods. We synthesized data sets for networks with 20 nodes. For each data set, we first randomly generated a valid network topology 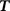. This step proceeds as follows. For each node, we selected the number of its parent from 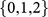, with probabilities with sum of 1. Once this number is determined, we chose the parents from the remaining nodes at random. This operation is applied to each node, which results in a full network candidate. Finally we checked the validity of the resulting network by checking whether there are directed loops. This network is used in the subsequent step if it passes the validity checking. Otherwise we repeated this process till a valid network topology is obtained. Once 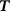 is known, we then randomly assigned a Boolean function to each node from all possible candidate functions, depending on its parent set. Thus we generated 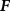. For 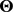, we randomly sampled these probability parameters from their prior distributions. Finally, with the generated 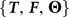, we applied Equation 1 to generate 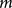 observations of the network 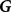. Since our model covers all possible boolean relationships with in-degree up to 2, the simulated data should be general enough for a fair comparison among BFE, REVEAL and TDBN.

To measure the inference accuracy, we define the correct rate (CR) as the percentage of the 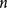 nodes whose parent sets and associated functions are both correctly identified as compared to the truth. Hence CR = 1 if and only if the inferred network indexed by 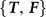 is the same as the true model.

To test BIBN, we synthesized different data sets with varying settings. The sample sizes tested include 50, 100, 300, and 500. The noise levels at 0.1 and 0.2 are considered. For each sample size and noise level combination, 20 different data sets corresponding to 20 different networks are generated. For each data set, a Markov chain is run with a total of 20,000 iterations. The first 15,000 iterations are treated as burn-in and the last 5,000 iterations are collected to calculate the average accuracy for a single chain. We averaged the 20 accuracies to obtain the final average accuracy for a specific sample size and noise level combination.

For comparison, we chose REVEAL and BFE which are two popular inference algorithms for Boolean network inference, and TDBN which is a recently developed method for reconstructing Boolean networks. Both REVEAL and BFE are implemented in the R package BoolNet [11]. The code of TDBN is from the author of [14]. The same data sets are inputted into REVEAL, BFE, TDBN and BIBN to obtain their inference accuracies. The results are summarized in Table 2. REVEAL is not listed in this table because its performance is very poor due to its low capability to handle nondeterministic network models. BFE and TDBN have a better tolerance of noise compared to REVEAL, but they are poor in pursuing the global optimization of the full network, thus resulting in lower correct rates. Obviously, Table 2 shows that our method outperformed all other methods for all settings. Generally speaking, when fixing the sample size, increasing noise level will deteriorate the inference accuracy. One can improve the accuracy by increasing the sample size when the noise level can not be reduced.

**Table 2:** Average accuracy comparisons on the synthesized data.

	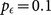			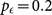		
Sample Size	BIBN	BFE	TDBN	BIBN	BFE	TDBN
10	0.1827	0.1725	0.1750	0.0809	0.0375	0.1425
50	0.8599	0.6975	0.4175	0.6858	0.5575	0.3300
100	0.9565	0.7425	0.4900	0.8864	0.7375	0.4375
300	0.9951	0.8575	0.7700	0.9358	0.8350	0.6800
500	1.0000	0.8775	0.8125	0.9975	0.8725	0.7825

It should be noted that TDBN calculated p values for all possible transition relations. We selected their most likely one to calculate the correct rate for comparison.

To further evaluate the proposed method, we also checked the prediction power of BIBN, with the results summarized in Table 3. In each scenario, we generated an observed sample as described before. Then we randomly chose 2/3 of the sample to perform the inference as we presented before, and the remaining 1/3 of the sample to test the prediction accuracy. More specifically, for each inferred network, we predicted the value of each child node using the observed values of its parents, then checked whether the predicted and observed values of the child are the same. The percentage of correct prediction over the 1/3 sample is treated as the prediction accuracy of this child node. The average prediction accuracy over all child nodes is treated as the prediction accuracy of whole network. This is done for the inferred network at each iteration after the burn-in period. The average prediction accuracy of these networks is treated as the prediction accuracy for this chain. This procedure is repeated independently for ten times for each scenario on Table 3. The correct prediction rate reported under each scenario in Table 3 is the average over the ten repetitions. It shows that BIBN has good prediction accuracy. Given the sample size, the correct prediction rate decreases as the noise level increases. With the noise level fixed, the correct prediction rate is improving as the sample size grows, which is as expected.

**Table 3:** Correct prediction rate of BIBN under difference scenarios.

Sample Size	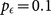	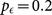
75	0.8877	0.7813
150	0.8929	0.7982
450	0.8977	0.8050
750	0.9149	0.8073

### Real Data Analysis

#### Cell-Cycle Gene Expression Data

The cell cycle is the biological process by which one cell grows and divides into two daughter cells. Due to its fundamental importance in cell biology, it has been studied extensively in various model organisms [25]–[28]. But due to its complexity, the complete composition and regulatory mechanisms of the cell-cycle gene network is still unclear for most eukaryotes.

Some studies indicate that components may vary over a long evolutionary distance [29]. However, most key components and their interactions are conserved [30]–[32]. With the cumulated gene expression data for yeast, we target at inferring the relationships among the key genes in yeast cell cycle.

Similar to the cell-cycle network used in [27], we study 14 key cell-cycle genes, including 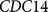, 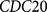, 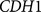, 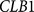, 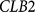, 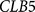, 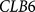, 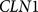, 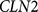, 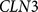, 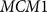, 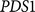, 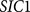 and 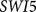. The real gene expression data can be downloaded from 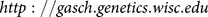. It contains the normalized data from 500 yeast microarray experiments under various conditions, including stress responses, cell-cycle synchronization, sporulation, etc. Missing values in the downloaded data are deleted since our current method only handles complete data. To transform the data into binary values, values that are higher than the corresponding gene's mean value are set to 1. Otherwise they are set to 0.

#### Network Inference Result

**Figure 1:**
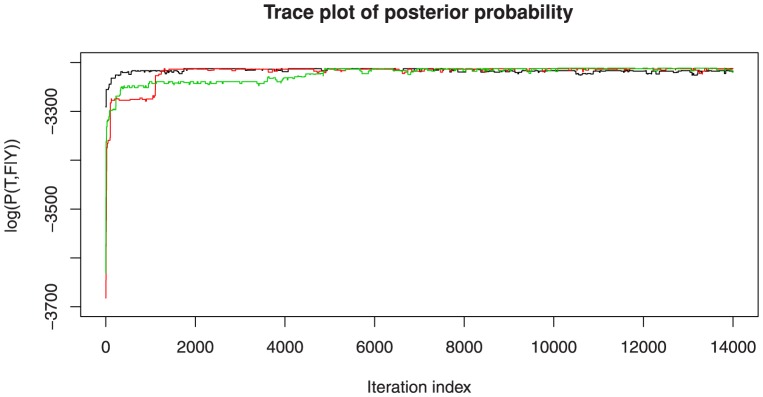
Trace plots of the unnormalized log-posterior probability of the Markov chain for real cell-cycle data. Each line represents an independent Markov chain. Each chain is run for 14,000 iterations.

For the transformed binary data set of 14 genes, we ran three independent Markov chains using three different initial networks, which include the empty network without any links, one randomly generated valid network and a valid network constructed from the preferential candidates. Each chain is run for 14,000 iterations. The trace plot of the unnormalized log-posterior probabilities for these three chains are displayed in Fig. 1. It shows that the chains converged after about 10,000 iterations. Thus, the network samples within the last 4,000 iterations are used for posterior inference.

It turns out that the last 4,000 iterations contain 43 unique network models. A total of 12.82% of the links in the reference yeast cell cycle network reported in [27] are identified in 100% of the posterior samples. For instance, the relation 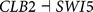 has a probability of over 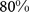 of being inferred correctly. The “coupled” gene pairs in [27], such as 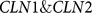, 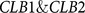 and 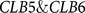, are correctly linked together in most of the posterior samples. Other correctly inferred relations also have a high show-up frequency in the posterior samples.

We also applied REVEAL, BFE and TDBN to this real data. The read data is too noisy for REVEAL and BFE to produce anything. While the accuracy of TDBN is 5.13%, which is much lower than that of BIBN. This comparison on real biological data clearly showed the advantage of our method, but it has be to admitted that we still need to improve our method in order to meet the accuracy requirement of real gene network inference. Future works shall check whether the boolean formulation is sufficient and whether the number of parents is not small for real biological network.

## Discussion

In this paper, we propose a new method for inferring the Boolean network from noisy data using a probabilistic model and an MCMC algorithm. Our inference focuses not only on the network structure but also on the transition functions associated with the network of interest. Compared to other inference algorithms, our method has the advantage of taking both random noise and model uncertainty into consideration, which is verified by the consistently higher inference accuracy for networks with varying sample size and noise levels in the simulation study. Furthermore, a data-based proposal is constructed using a 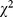 goodness-of-fit test for guiding the proposal of new local topology and function relations. Since the search space of networks is so large, especially for networks with many nodes, the use of carefully chosen proposals greatly improves the inference efficiency in terms of the fewer iterations needed to reach the convergence of the chain. Currently our algorithm, which is implemented in R and run on a 2.66 GHz CPU, takes about 1.6 hours to run 20,000 iterations when the sample size is 50, and 1.9 hours when the sample size is 500.

It should be noted that our method also has some limitations. One is the assumption that each node has at most two parents, which may limit its wide application in practice. In principle, the method can be extended to deal with networks with more than 2 parents for each node without further technical difficulties. However, the computational requirements of the method would increase significantly and there is a danger to overfit the data. Another shortcoming of our method is to assume the model to be a directed acyclic graph in order to use the Bayesian network framework [33]. Regulatory networks are known to contain feedback loops, thus our inference shall be considered as a preliminary step. Future research can extend our model on the line of dynamic Bayesian network in order to model loops [34]. Also, since our method is based on Boolean values, genes with more than two expressing status or gene relations may not be correctly modeled here. The method for discretizing gene expression values is also a very important issue and deserves the exploration of a separate paper [35]. In terms of future enhancement, techniques for MCMC algorithms to avoid trapping in local modes can be added.
